# Vitamin D Status in Central Europe

**DOI:** 10.1155/2014/589587

**Published:** 2014-03-26

**Authors:** Pawel Pludowski, William B. Grant, Harjit Pal Bhattoa, Milan Bayer, Vladyslav Povoroznyuk, Ema Rudenka, Heorhi Ramanau, Szabolcs Varbiro, Alena Rudenka, Elzbieta Karczmarewicz, Roman Lorenc, Justyna Czech-Kowalska, Jerzy Konstantynowicz

**Affiliations:** ^1^Department of Biochemistry, Radioimmunology and Experimental Medicine, The Children's Memorial Health Institute, Aleja Dzieci Polskich 20, 04-730 Warsaw, Poland; ^2^Sunlight, Nutrition and Health Research Center, San Francisco, CA 94164-1603, USA; ^3^Department of Laboratory Medicine, Medical and Health Science Center, University of Debrecen, Debrecen 4032, Hungary; ^4^Department of Pediatrics, Charles University Prague, Faculty of Medicine in Hradec Kralove, University Hospital Hradec Kralove, 500 05 Hradec, Czech Republic; ^5^D.F. Chebotarev Institute of Gerontology of National Academy of Medical Sciences of Ukraine, Kiev 04114, Ukraine; ^6^Belarusian Medical Academy of Postgraduate Education, 220013 Minsk, Belarus; ^7^Internal Medicine of Gomel Medical University, Gomel, Belarus; ^8^2nd Department of Obstetrics and Gynecology, Faculty of Medicine, Semmelweis University, Budapest 1082, Hungary; ^9^Cardiology and Rheumatology of Belarusian Medical Academy of Postgraduate Education, 220013 Minsk, Belarus; ^10^Department of Neonatology and Neonatal Intensive Care, The Children's Memorial Health Institute, 04 730 Warsaw, Poland; ^11^Department of Pediatrics and Developmental Disorders, Medical University of Bialystok, 15 274 Bialystok, Poland

## Abstract

Little published information is available regarding epidemiological data on vitamin D status in the large geographical region of Central Europe (CE). We searched the journal literature with regard to 25(OH)D concentrations among community-dwelling or healthy people living in CE. 25(OH)D concentrations varied by age, season, study sample size, and methodological approach [i.e., 25(OH)D assay used]. Concentrations of 25(OH)D in CE appeared lower than 30 ng/mL, and the magnitude of hypovitaminosis D was similar to that reported in Western Europe. While most of the studies reviewed were cross-sectional studies, a longitudinal study was also included to obtain information on seasonal variability. The longitudinal study reported wintertime 25(OH)D values close to 21–23 ng/mL for all studied age groups, with a significant increase of 25(OH)D in August reaching 42 ng/mL for those aged 0–9 years, but only 21 ng/mL for the elderly aged 80–89 years. The decrease in 25(OH)D with respect to age was attributed to decreased time spent in the sun and decreased vitamin D production efficiency. Based on the literature review on vitamin D status in the CE populations, it can be concluded that 25(OH)vitamin D levels are on average below the 30 ng/mL level.

## 1. Introduction

The literature published over the two last decades indicates increasing awareness of vitamin D's pleiotropic, multidirectional action in the human body. Evidence from large-scale studies contributed to the understanding that vitamin D deficiency may be a significant risk factor for many civilization diseases. There is recognized benefit of vitamin D for bone health based on both observational studies and randomized controlled trials [1]. There is also evidence largely from cross-sectional, ecological, laboratory, and observational studies that vitamin D reduces risk of many types of cancer, cardiovascular disease, diabetes, autoimmune and metabolic disorders, infectious diseases linked to decreased immunity, and even some neuropsychiatric disorders [2–8]. Based on the journal literature for the nonskeletal effects of vitamin D, it appears that serum 25-hydroxyvitamin D [25(OH)D] concentrations between 30 and 50 ng/mL are associated with significantly reduced risk of such diseases [9–12]. Therefore, a variety of practical and research activities are being undertaken worldwide to evaluate vitamin D deficiency and improve vitamin D status. In Central Europe (CE), researchers representing the region developed recommendations to treat vitamin D deficiency for Poland in 2009 [13] and for Hungary in 2012 [14]. Because of convincing findings showing potential health benefits of vitamin D, investigators in CE focus on determining serum 25-hydroxyvitamin D [25(OH)D] concentrations in the general population and among different risk groups. This interest inspired a conference, “Vitamin D—minimum, maximum, optimum,” held in Warsaw, Poland, on October 19–20, 2012 (http://www.witaminad.waw.pl/). The meeting was organized by the Children's Memorial Health Institute, Department of Biochemistry, Radioimmunology, and Experimental Medicine, in Warsaw, with 550 attendees from European and non-European countries. The conference sought to establish recommendations on serum 25(OH)D concentrations for Central Europeans. A related goal was to develop an understanding of current serum 25(OH)D normative ranges and of how they vary with respect to such factors as age, sex, and season. The major purpose was to establish guidelines for appropriate vitamin D supplementation for Central Europeans of all ages in order to ensure adequate serum 25(OH)D concentration and, thereby, to guarantee short- and long-term effects, with appropriate safety considerations. The primary conclusion reached by the participants at the Warsaw conference was consensus on optimal (target) serum 25(OH)D concentrations ranging from 30 to 50 ng/mL (75–125 nmol/L). Although no convincing reports indicate adverse health effects of serum 25(OH)D concentrations up to 100 ng/mL (250 nmol/L), few studies show health benefits associated with levels higher than 50 ng/mL.

## 2. Materials and Methods

This paper reviews the available spectrum of data on serum 25(OH)D concentrations in CE, compared with selected findings from other European countries. We found several articles through advanced searches of the National Library of Medicine's PubMed database and Scopus, using keywords “vitamin D” or “serum 25-hydroxyvitamin D” along with country names or “Europe.” Some of the CE “epidemiologic” studies reported at the vitamin D conference in Warsaw were also included for further analyses. Papers dealing with healthy or community-dwelling people were included in the tables, but people with diseases were not. However, one set of data for patients was given in a separate table because it provided longitudinal data on serum 25(OH)D concentrations throughout the year [15].

## 3. Results

Tables 1–4 provide explicit comparative information on serum 25(OH)D concentrations in Central European countries as a function of age [16–46], whereas Table 5 gives information as a function of season (monthly intervals) stratified by age for a Hungarian population [15].

### 3.1. Neonates and Infants

Eight studies in this review reported serum 25(OH)D concentrations for neonates and infants in CE: one from the Czech Republic and seven from Poland (Table 1). Mean serum 25(OH)D concentration among neonates ranged between 7 and 24 ng/mL depending on season. Winter and spring values were low, 7–14 ng/mL, whereas summertime values were better (19–24 ng/mL). Recent Polish studies confirmed the above observations, showing higher summertime than winter/spring mean 25(OH)D concentrations in the umbilical cord: 24.0 ± 8.5 ng/mL versus 13.5 ± 8.2 ng/mL (*P* < 0.001), respectively [20–22]. Serum 25(OH)D values found in these studies appeared lower than those recommended on the basis of a recent randomized controlled trial of vitamin D supplementation during pregnancy. This study, performed by Hollis and colleagues, demonstrated association between the 25(OH)D level of 40 ng/mL and optimal serum 1,25-dihydroxyvitamin D concentrations [47]. Fortunately, implementing recommendations for neonates to start vitamin D supplementation from the first days after delivery resolved, at least partly, vitamin D deficiency during the first few months of life. As Czech-Kowalska and colleagues showed, supplementing neonates with daily doses of ~550 IU of vitamin D increased serum 25(OH)D to 55 ng/mL at the third month of life [22]. Further, in the group of infants (*n* = 43) regularly supplemented with a vitamin D dose of ~1160 IU/day at both the 6th and 12th month, 25(OH)D serum concentrations unexpectedly decreased from 40.2 ± 18.8 ng/mL at the 6th month to 32.0 ± 12.7 ng/mL at the 12th month (*P* < 0.01) [17]. However, reduced daily vitamin D intake expressed in international units/kilogram of body weight may account for the observed decrease in 25(OH)D concentration [23].

### 3.2. Children and Adolescents

Table 2 shows serum 25(OH)D concentrations in children and adolescents. In Central European countries, wintertime values ranged from 9 ng/mL in Belarus [24] to 23 ng/mL in Hungary [25]; summertime values ranged from 36 to 56 ng/mL. The large winter range may be due to different 25(OH)D assays used, which will be discussed later. In addition, studies with smaller sample size may have been associated with variations in 25(OH)D concentrations due to recruiting people who may not have been representative of the larger population.

### 3.3. Adults

Table 3 presents serum 25(OH)D concentrations for adults aged 20–60 years. In CE, wintertime 25(OH)D concentrations ranged from 11 ng/mL in Poland to 18 ng/mL in Estonia. Summertime 25(OH)D concentrations ranged from 18 ng/mL in Ukraine to 35 ng/mL in Hungary, and annual values found in larger studies (>100 cases) ranged from 14 ng/mL in Ukraine to 29 ng/mL in Belarus. In Western European countries of similar latitude, wintertime values ranged from 13 ng/mL in Denmark to 20 ng/mL in Austria, whereas those in summertime ranged from 23 to 35 ng/mL, and annual values were reported as 25 ng/mL in France [41]. Thus, 25(OH)D serum concentrations of Central European and Western European countries showed consistent agreement. Some information is available in the studies regarding serum 25(OH)D concentrations in men and women. A study from Great Britain involving 45-year olds in a cohort study found that women had statistically higher concentrations than men in winter, while men had statistically higher concentrations in summer [42]. The differences might be due to men spending more time outdoors and women taking more oral vitamin D. A study from Estonia found similar but statistically nonsignificant results: in summer, men had a mean serum 25(OH)D concentration of 24.2 ng/mL while women had 23.4 ng/mL, while in winter the values for males and females were 17.1 ng/mL and 17.8 ng/mL, respectively [32].

### 3.4. The Elderly

Table 4 gives serum 25(OH)D concentrations for seniors aged 60 years or older. In Central European countries, wintertime 25(OH)D concentrations ranged from 11 ng/mL in Ukraine to 20 ng/mL in Hungary. Summertime 25(OH)D concentrations ranged from 15 ng/mL in Ukraine to 33 ng/mL in Hungary. Annual 25(OH)D concentrations ranged from 13 ng/mL in Ukraine to 29 ng/mL in Hungary. In Western European countries, wintertime values ranged from 17 to 20 ng/mL. Analyzing serum 25(OH)D concentrations with respect to latitude in either Central or Western European countries revealed no consistent variability. At least in part, the reasons for this could include that the solar ultraviolet-B (UVB) dose gradient during European summer is not large above 40°N latitude and that skin pigmentation becomes lighter as latitude increases, making it easier to generate vitamin D from solar UVB [48]. As noted in Table 5, serum 25(OH)D concentrations decrease with age above about 50 years. Since most studies summarized in this table reported 25(OH)D concentrations for a limited range of ages, stated in the table, the values in the table should be considered representative of those for the age ranges studied and not for those over the age of 60 years.

### 3.5. Effect of Age and Season

A useful study on the variation of serum 25(OH)D3 concentration with respect to age in 10-year groupings and month of measurement (Table 5) was reported for a population from Budapest, Hungary (47.5°N latitude, 16.8°E longitude) [15]. Although the subjects studied were patients, nothing indicated that their morbidity affected serum 25(OH)D3 concentration. However, the report noted that, for the 1307 subjects with repeated measurements, serum 25(OH)D concentrations were lower for the second measurement (26 ± 9 ng/mL) than for the first (27 ± 13 ng/mL), suggesting that the medical staff did not recommended taking vitamin D supplements. Table 5 gives a summary of data from that study. Several months were omitted for which serum 25(OH)D3 concentrations either did not change or were inconsistent with concentrations for other months; values in January were similar to those in May. Several associations become clear from the content of Table 5: serum 25(OH)D3 concentration increased minimally before June except for the population aged 0–9 years. For all ages, serum 25(OH)D3 concentration started to decline in September and reached wintertime values by October. Peak serum 25(OH)D3 concentrations were the highest for the youngest people and the lowest for the oldest people. The wintertime mean serum 25(OH)D concentration was about 20–23 ng/mL for all ages. The increase in summer amounted to 20 ng/mL for those aged 0–9 years, 14–15 ng/mL for those aged 10–49 years, 10 ng/mL for those aged 50–69 years, and 5–6 ng/mL for those aged 70–89 years. Two primary factors accounted for age-related seasonal fluctuations (i.e., differences in summertime peak values): limited time spent outdoors in sunlight and reduced efficiency of vitamin D production from UVB irradiance. In a mid-1980s study, vitamin D production efficiency reported for people older than 60 years was about 25% of that for those younger than 20 years [49], owing to less 7-dehydrocholesterol in the skin, which is converted to vitamin D3 through the action of UVB irradiance followed by a thermal process. The change in vitamin D production in summer as a function of age agrees with the efficiency study. Those with darker skin make vitamin D more slowly than those with light skin since the melanin in the skin reduces the transmission of solar UVB to the 7-dehydrocholesterol. In addition, Table 5 gives calculated standard vitamin D doses (SDD) for whole-day irradiance for solar UVB measured in Belsk, Poland (52°N latitude, 21°E longitude) [50]. However, because vitamin D3 production is limited to 10 000–20 000 IU/day (since UV both produces vitamin D and destroys its metabolites), one cannot use the SDD values to estimate vitamin D production for a given time in the sun. For such information, the graphs in the papers by Webb and Engelsen [51] and Bakos and Mikó [52] are useful. Vitamin D production potential peaks near the end of June, whereas serum 25(OH)D3 concentration peaks in August. The lag of about 6 weeks is related primarily to the time required to build up serum 25(OH)D concentration. Serum 25(OH)D is the most important clinically available measurement of vitamin D status, reflecting lifestyle and dietary habits [53]. Determining the amount provided by the sun or food is difficult. The duration and intensity of exposure to sunlight are not easily measurable, and age, skin pigmentation, sunscreens, clothing, and even window glass reduce its effects [54]. In equatorial regions exposure to the sun alone is adequate, but at latitudes above 40 degrees north or south and higher, people make little vitamin D in the winter. Measurement of serum 25(OH)D provides direct information. Although its concentration depends on vitamin D production and intake, its serum half-life is much longer than that of vitamin D (weeks versus hours), and it therefore provides an integrated assessment of vitamin D status. Serum 25(OH)D concentrations depend on age, sunlight exposure, vitamin D dietary intake, or supplementation.

### 3.6. 25(OH)D Assays Used

The spectrum of methods commonly used in research and laboratory practice includes three types: manual immunoassays, automated immunoassays, and direct detection methods. Most instruments or approaches yield reasonably accurate measurements; however, some instruments appear problematic [44]. Several reports have also discussed analogous pitfalls of the assays [55–59]. In a comparison of 25(OH)D assays in Sweden, a high-pressure liquid chromatography (HPLC) assay measured 34 ± 2 ng/mL, a radioimmunoassay (RIA) measured 28 ± 2 ng/mL, and a competitive immunochemiluminescence assay (CILA) measured 24 ± 2 ng/mL [56]. In a comparison of assays with liquid chromatography-tandem mass spectrometry methods in Australia, DiaSorin LIAISON, IDS, and Siemens assays met minimum performance goals [59]. In a comparison study in Warsaw, the Elecsys (total vitamin D) from Roche measured about 2 ng/mL higher than the LIAISON from DiaSorin [60]. Immunoassays are sensitive to 24,25-dihydroxyvitamin D, which can occur at concentrations up to 5 ng/mL [61]. Vitamin D-binding protein concentrations also affect the accuracy of serum 25(OH)D concentration measurement [62]. Some laboratories validated their assay performance by comparing measurements with samples submitted to the international Vitamin D External Quality Assessment Scheme (DEQAS) [58]. Comparability of 25(OH)D results could be facilitated if all laboratories were to participate with DEQAS.

## 4. Discussion

To our knowledge, this study is the first to summarize available data regarding vitamin D status and epidemiology in Central European populations of different ages. Most populations and most age groups have at least a moderate deficit of 25(OH)D according to currently binding standard references. The potential limitation we acknowledge is that all studies in this review are either retrospective or cross-sectional. To draw firm conclusions on intraindividual variations in 25(OH)D levels in different seasons, a prospective study design would be desirable. With the exception of two studies [43, 44], no particular inclusion or exclusion criteria for study participation were assumptive; therefore, we recognize that studied populations may have been heterogeneous. Furthermore, 25(OH)D3 and total 25(OH)D concentrations were usually similar but not identical, so we analyzed results from studies irrespective of type of vitamin D determination. A review of 394 studies of unadjusted serum 25(OH)D concentrations from around the world found a mean value of 22 ± 1 ng/mL, with no effect of latitude for nonwhites [63]. However, the regression fit to the data for white people went from approximately 40 ng/mL near the equator to approximately 16 ng/mL at the poles. What happens in Europe is still not clear from that paper. Evidently, skin pigmentation (as well as diet at high latitudes) have adapted well to solar UVB doses where people have lived for millennia [48]. A review of serum 25(OH)D concentrations among dark-skinned people living in Europe—primarily those of African, Asian, or Middle Eastern origin—supports this hypothesis. These ethnically different groups had lower serum 25(OH)D concentrations than the indigenous white inhabitants [64]. The three important factors contributing to the difference were darker skin, clothing that covered more skin area, and limited oral vitamin D intake from food. Serum 25(OH)D concentrations in winter do not drop as low as might be expected on the basis of solar UVB doses in winter for two reasons: (1) the decay time of 25(OH)D is 4–6 weeks—that is, the time it takes to drop to half its value—and (2) when serum 25(OH)D concentrations are low, the body converts vitamin D to 25(OH)D much more efficiently [65].

The following question emerges: if the natural sources of vitamin D that arrived at over millennia lead to mean annual serum 25(OH)D concentrations slightly above 20 ng/mL, why is this value not adequate? One point to be addressed is that life expectancy has considerably increased in Europe and elsewhere during the past century because of health care advances that reduced the risk of dying from accidents, digestive diseases, and respiratory and other infections [66]. Europeans are therefore much more likely to die now from cancer or cardiovascular disease. Ecological and observational studies offer moderate evidence that vitamin D reduces the risk of cancer [67–69] and cardiovascular disease [70]. Thus, raising serum 25(OH)D concentrations above 30–40 ng/mL should reduce mortality rates by about 15% and increase life expectancy by 2 years in Europe [71]. Although the above associations may be regarded cautiously and require further long-term prospective investigation, it is rather justified to recommend an individualized vitamin D supplementation to all age groups in CE. The practical approach of such a strategy is aimed to alleviate the vitamin D status in this region—that is, to consequently diminish the risk of 25(OH)D deficits.

## 5. Summary and Conclusion

The essential finding in this review is that most people living in both Central and Western Europe have serum 25(OH)D concentrations below the optimal values of 30–50 ng/mL. The main reason is that solar UVB, being the primary source of vitamin D, is limited for most CE populations; thus, producing vitamin D from solar UVB from October through March is nearly impossible above 40°N latitude. By consequence, the concentrations are particularly low from October through May, implicating the deficiency to a large extent [15]. Also, most people spend most time indoors and so they produce vitamin D only through casual sunlight exposure, which raises mean serum 25(OH)D concentration from 15 ng/mL in February to 30 ng/mL in September for individuals aged 45 years living in the UK [42]. The groups at particularly high risk of vitamin D deficiency include those largely staying indoors, pregnant and nursing women, newborns, breast-fed infants without vitamin D supplementation, overweight or obese people [72], patients with chronic or infectious disease, and those older than 50 years. A variety of preventive means and interventions can be implemented in CE to increase serum 25(OH)D concentrations, including increased but reasonable solar UVB irradiance, fortification of food, and augmented consumption of vitamin D supplements.

## Figures and Tables

**Table 1:** Serum 25-hydroxyvitamin D concentrations reported for neonates and infants. (Mean, range, and standard deviations are shown.)

Country	City	Latitude, longitude	Year	Number, sex	Age	Population	Season	Assay, machine (manufacturer)	Serum 25(OH)D (ng/mL)	Reference
Czech Republic	Pilzen	49.8°N 13.3°E	April–June 2006	28	Newborn	Term, cross section	Spring	CLIA, Liaison (DiaSorin)	7 (6–13)	[16]

Poland	Warsaw	52.2°N 21.0°E		31	Newborn	Healthy	Winter	Radiocompetitive, Extrelut column and radioassay	7 ± 5	[17]
Poland	Warsaw	52.2°N 21.0°E		22	Newborn	Healthy	Summer		19 ± 10	[17]

Poland	Warsaw	52.2°N 21.0°E	2001-2002	20 M 17 F	Newborn 1 week	Healthy	Annual	CLIA, Liaison (DiaSorin)	15 ± 9	[18]

Poland	Warsaw	52.2°N 21.0°E	2001-2002	56	Newborn 3 weeks	Healthy	Annual	CLIA, Liaison (DiaSorin)	15 ± 9	[19]

Poland	Warsaw	52.2°N 21.0°E		76	Newborn cord blood	Healthy	Winter	CLIA, Liaison (DiaSorin)	14 ± 8	[20]

Poland	Warsaw	52.2°N 21.0°E		40	Newborn cord blood	Healthy	Summer	CLIA, Liaison (DiaSorin)	24 ± 9	[21]

Poland	Warsaw	52.2°N 21.0°E		15 M 15 F	2 weeks	Healthy	Winter, summer	CLIA, Liaison (DiaSorin)	8.5 (7–12)	[22]

Poland	Warsaw	52.2°N 21.0°E		15 M 15 F	10 weeks	Healthy, after supplementation	Winter, summer	CLIA, Liaison (DiaSorin)	55 (35–67)	[22]

Poland				134	6 months	Healthy, after supplementation		RIA	43 ± 20	[23]

Poland				98	12 months	Healthy, after supplementation		RIA	29 ± 12	[23]

**Table 2:** Serum 25-hydroxyvitamin D concentrations reported for children and adolescents.

Country	City	Latitude, longitude	Year	Number, sex	Age (yrs)	BMI	Population	Season	Assay, machine (manufacturer)	Serum 25(OH)D (ng/mL)	Reference
Belarus	Minsk	53.9°N 27.6°E	2011-2012	47 M 33 F	11 (8–13)		Healthy	Autumn-winter	ECLIA, Cobas e411 (Roche Diagnostics)	9 (5–15)	[24]

Hungary	Budapest	47.5°N, 17.1°E		100 M	11–14	20	Healthy, Cross section	Winter	CLIA, IDS (IDS)	23 ± 6	[25]
Hungary				66 M	11–14	20	Healthy	Summer		41 ± 13	
Hungary				91 F	11–14	20	Healthy	Winter		21 ± 8	
Hungary				53 F	11–14	20	Healthy	Summer		38 ± 14	

Poland		49–54°N 15–24°E		199 F	13 ± 1		Community, cross section	Winter	HPLC	12	[26]

**Table 3:** Serum 25-hydroxyvitamin D concentrations reported for adults in Central Europe.

Country	City	Latitude, longitude	Year	Number, sex	Age (yrs)	BMI	Population	Season	Assay, machine (manufacturer)	Serum 25(OH)D (ng/mL)	Reference
Belarus	Western Belarus	53°N 24–26°E	2010-2011	6 M 22 F	46 ± 7	27 ± 4	Healthy	Annual	ECLIA, Elecsys (Roche Diagnostics)	18 ± 7	[28]

Belarus	Minsk		2011-2012	168 F	45–55		Healthy	Annual	ECLIA, Cobas e411 (Roche Diagnostics)	29 ± 15	[29]
Belarus	Minsk			176 F	55–65			Annual		27 ± 14	[29]

Czech Republic	Prague	50.1°N 14.4°E	2004–2006	2175			Clinic patients	Annual	RIA, IDS, UK	31 ± 18	[30]

Czech Republic	Pilzen	49.8°N 13.3°E	2008	239 M 321 F	53 ± 14	27 ± 5	Community, cross section	Oct. 6–Nov. 28	ECLIA, Cobas e411 (Roche Diagnostics)	25 ± 4	[31]

Estonia	Väike-Maarja	59.1°N 26.3°E	2006	167 M	49 ± 12	28 ± 5	Community, cross section from patients	Winter	RIA, DiaSorin	17 ± 6	[32]
Estonia				200 F	49 ± 12	29 ± 7	Community	Winter		18 ± 6	[32]
Estonia				167 M	49 ± 12	28 ± 5	Community	Summer		24 ± 7	[32]
Estonia				200 F	49 ± 12	29 ± 7	Community	Summer		23 ± 7	[32]

Hungary	County Vas	47.2°N 16.8°E	2011	32 M	<43		Healthy blood donors and others, cross section	March–May	ECLIA, Cobas e411 (Roche Diagnostics)	29 (25–40)	[33]
Hungary	County Vas			48 F	<43		Healthy			22 (29–40)	[33]
Hungary	County Vas			36 M	>43		Healthy			27 (22–34)	[33]
Hungary	County Vas			21 F	>43		Healthy			24 (16–34)	[33]

Poland	Warsaw	52.2°N 21.0°E		31	Mothers at delivery			Winter	Radiocompetitive, Extrelut column and radioassay	11 ± 7	[17]
Poland	Warsaw			22	Mothers at delivery			Summer		31 ± 15	[17]

Poland	Katowice	50.3°N 19.0°E	2003-2004	17 F	52 ± 4	24 ± 2	Healthy	Annual	RIA, Bio-Source Europe	39 ± 18	[34]

Poland	Opole	50.6°N 17.9°E		31 F	47 (25–79)		Healthy, employees of the Center of Oncology, Opole	November–March	ECLIA, (Roche Diagnostics)	17	[35]

Poland	Warsaw			76	Mothers after delivery		Healthy	Winter	CLIA, Liaison (DiaSorin)	15 ± 8	[20, 21]
Poland	Warsaw			40	Mothers after delivery		Healthy	Summer		20 ± 7	[20, 21]
Poland	Warsaw			119	Lactating women		Healthy	Annual		26 ± 7	[20, 21]

Poland	Warsaw			138 F	Pregnant women 1st trimester		Healthy	Annual		17.6 (4–57)	[36]

Poland	Warsaw			138 F	Pregnant women 3rd trimester		Healthy	Annual		18.5 (4–40)	[36]

Poland	Warsaw			55	Pregnant women 1st trimester		Healthy	Annual	ECLIA, Elecsys 2010 (Roche Diagnostics)	23 (17–57)	[37]
Poland	Warsaw			55	Pregnant women 2nd trimester		Healthy	Annual		25 (6–53)	[37]
Poland	Warsaw			55	Pregnant women 3rd trimester		Healthy	Annual		25 (3–50)	[37]

Slovakia		49°N 17–22°E	2007	162 F	34		Healthy	October	HPLC	33 ± 13	[38]

Ukraine		44.2°N–52.2°N	2010-2011	649 F	47 (20–59)	28 ± 6	Healthy	Annual	ECLIA, Elecsys 2010 (Roche Diagnostics)	14 ± 9	[39, 40]
Ukraine			2010-2011	129 M	44 (20–59)	26 ± 6	Healthy	Annual		15 ± 10	[39, 40]
Ukraine			2010-2011	102 F 28 M	47 ± 10	27 ± 5	Healthy	Winter		13 ± 8	[39, 40]
Ukraine			2010-2011	160 F 37 M	45 ± 11	27 ± 5	Healthy	Summer		18 ± 10	[39, 40]

**Table 4:** Serum 25-hydroxyvitamin D concentrations reported for seniors.

Country	City	Latitude, longitude	Year	Number, sex	Age (yrs)	BMI	Population	Season	Assay, machine (manufacturer)	Serum 25(OH)D (ng/mL)	Reference
Belarus				178 F	65–75			Annual	ECLIA, Cobas e411 (Roche Diagnostics)	26 ± 14	[29]
Belarus				101 F	>75			Annual		19 ± 9	[29]

Hungary	Debrecen	47.5°N 21.6°E		319 F	65 (41–91)	26 ± 4	Community	Year	RIA, DiaSorin	19 (5–54)	[43]
Hungary	Debrecen			100 F	65		Community	Spring		17 (5–40)	[43]
Hungary	Debrecen			80 F	65		Community	Summer		20 (5–41)	[43]
Hungary	Debrecen			79 F	65		Community	Autumn		21 (5–54)	[43]
Hungary	Debrecen			60 F	65		Community	Winter		20 (5–41)	[43]

Hungary	Debrecen	47.5°N 21.6°E	September 2009–September 2010	206 M	60 (51–81)	29 (17–42)	Healthy	Year	HPLC	29 (4–74)	[44]

Hungary	Debrecen			59 M	60	28	Community	Spring	HPLC	27 (4–66)	[44]
Hungary	Debrecen			96 M	61	30	Community	Summer		33 (7–74)	[44]
Hungary	Debrecen			24 M	61	29	Community	Autumn		25 (6–58)	[44]
Hungary	Debrecen			30 M	59	29	Community	Winter		23 (5–45)	[44]

Poland				65 F	72 ± 1		Healthy	Winter	HPLC	13	[26]

Ukraine				149 F	65	29 ± 5	Healthy, not treated with vitamin D, cross section	Winter	CLIA, Liaison (DiaSorin) and ECLIA, Elecsys 2010 (Roche Diagnostics)	13 ± 7	[39, 40]
Ukraine				124 F	75	30 ± 4	Healthy,	Winter		14 ± 8	[39, 40]
Ukraine		44.2°N–52.2°N 25–40°E	2010-2011	711 F	69 (60–95)	29 ± 5	Healthy	Annual		13 ± 8	[39, 40]
Ukraine			2010-2011	86 M	71 (60–91)	28 ± 4	Healthy	Annual		16 ± 9	[39, 40]
Ukraine			2010-2011	120 F	69 ± 6	30 ± 6	Healthy	Winter		11 ± 6	[39, 40]
Ukraine			2010-2011	305 F	68 ± 6	28 ± 5	Healthy	Summer		15 ± 8	[39, 40]

**Table 5:** Serum 25(OH)D_3_ concentration (ng/mL) versus age range and month measured for patients at Semmelweis University, Budapest, between April 2009 and March 2010 [9].

Month	0–9 years	10–19	20–29	30–39	40–49	50–59	60–69	70–79	80–89	SDD
March	25	23	23	23	22	22	23	23	20	4
May	31	23	25	24	23	24	24	21	21	21
June	30	26	30	30	28	26	29	28	21	56
July	35	30	33	31	28	27	27	25	19	55
August	42	37	35	35	36	29	33	25	21	42
September	36	30	30	29	30	29	26	26	16	18
October	31	23	23	27	24	24	23	20	15	9
November	23	23	25	26	27	23	27	26	23	2
December	22	22	21	21	21	19	23	20	15	1
